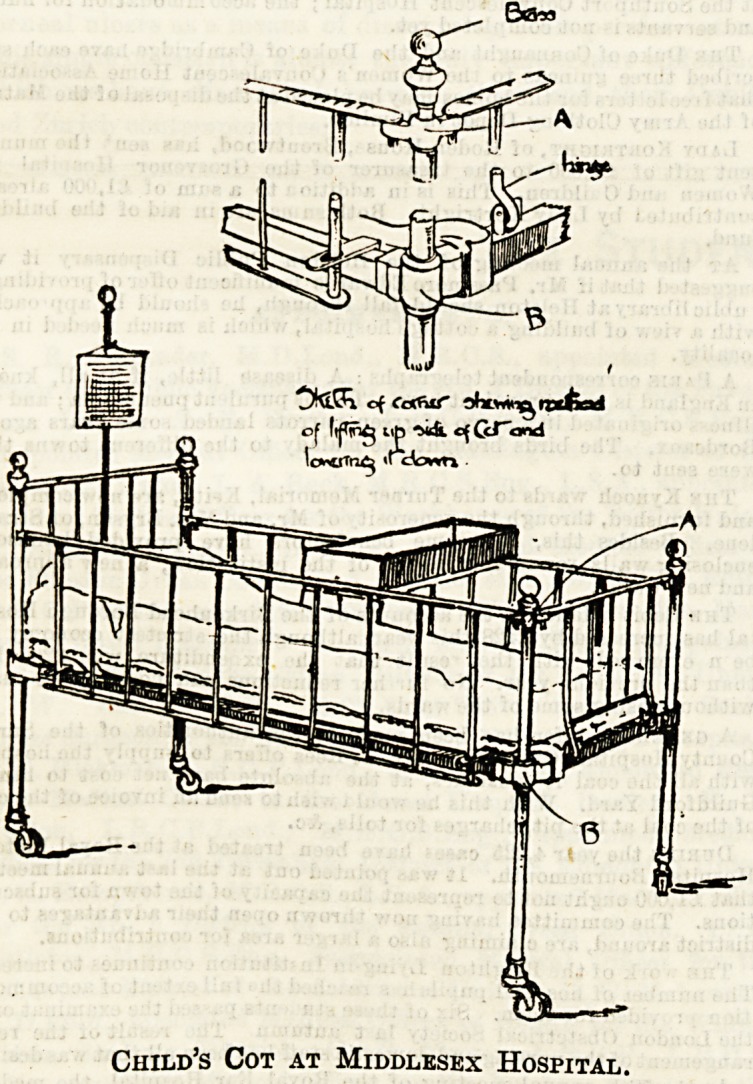# Children's Cots

**Published:** 1895-02-16

**Authors:** 


### PRACTICAL DEPARTMENTS.

### CHILDREN'S COTS.

Some weeks since we gave in these columns a sketch of a child's cot, with sliding sides, which was subsequently

COT AT POPLAR HOSPITAL FOR ACCIDENTS.

commented upon in a letter from Mr. Holland, the chairman of the Poplar Hospital for Accidents, who drew attention to the cots in the children's ward at that institution as being,

CHILD'S COT AT MIDDLESEX HOSPITAL.

in one or two details, better constructed. We are now able to give a drawing of the cot in question, from which it will be seen that the top side rail merely rests on a small hooked support in the side posts themselves, thereby avoiding the slight projection to which Mr. Holland objected as a possible source of damage to the little occupants or of inconvenience to the nurse. Certainly nothing could well be more simple than this particular make of cot. The principle is the same as in the one previously described, but the increased simplicity, and the minimising of corners is an improvement. Another point to b noted is that the lower rail of the side pieces is curved, so that when dropped upon the floor there is no danger to the toes of anyone standing beside the cot. Head and foot pieces are also removable, but only by the unscrewing of the corner knobs, when they may be lifted right away. It is naturally only occasionally that such a removal is necessary, therefore the same need for easy manipulation does not exist as for the sides, but the most modern cots are so constructed that all four sides can be removed by merely lifting up and either dropping down or turning over, as the case may be.

The second illustration given here shows one of the cots in use at the Middlesex Hospital fulfilling the above conditions. The two long side pieces are secured by a pin at either end, passing through a slot, much after the same fashion as in the first drawing, and slide down in the same manner also. The head and foot pieces lift up in the same way, but turn over with a hinge, and all unscrewing of knobs is avoided. In the drawing the method is clearly shown. Both cots are admirable in every way, and could hardly be improved upon. Those at Poplar were made by Messrs. Atkinson, the well-known firm in Westminster Bridge Road, and the Middlesex cots came from Messrs. Nettlefold and Sons, High Holborn, W.C.

Excellent children's cots are also to be seen at the Chelsea Infirmary, made, we believe, after the design of one of the doctors, by Messrs. Shoolbred, and similar in general character to those above described.

## Figures and Tables

**Figure f1:**
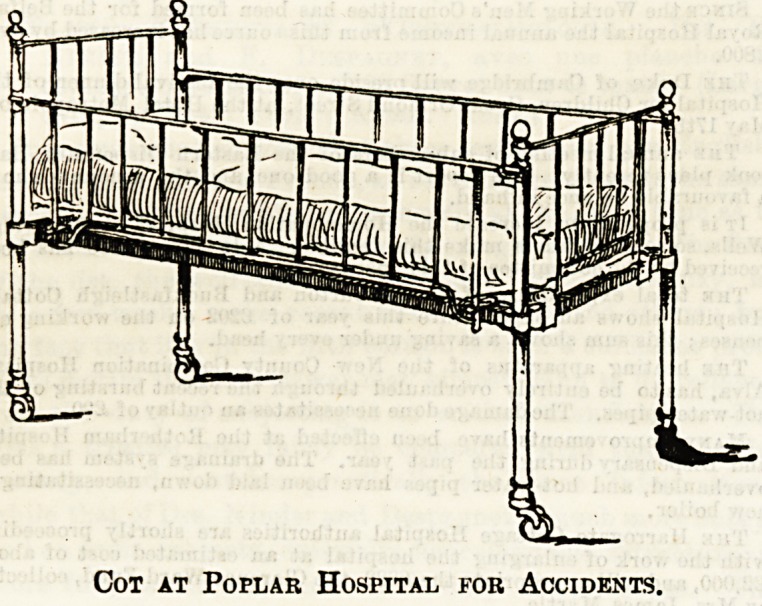


**Figure f2:**